# Playing nice: a multi-methodological study on the effects of social conformity on memory

**DOI:** 10.3389/fnhum.2013.00079

**Published:** 2013-03-18

**Authors:** Lorena Deuker, Anna R. Müller, Christian Montag, Sebastian Markett, Martin Reuter, Juergen Fell, Peter Trautner, Nikolai Axmacher

**Affiliations:** ^1^Department of Epileptology, University of BonnBonn, Germany; ^2^Department of Psychology, University of BonnBonn, Germany; ^3^Center for Economics and Neuroscience, University of BonnBonn, Germany; ^4^Department of NeuroImaging/NeuroCognition, Life and Brain Center, University of BonnBonn, Germany; ^5^German Center for Neurodegenerative Diseases, University of BonnBonn, Germany

**Keywords:** social conformity, fMRI, recognition memory, Catechol-O-methyl transferase (COMT), hippocampus, anterior cingulate cortex

## Abstract

Conformity is an important aspect of social behavior. Two main motives have been identified: people may adapt their behavior to “play nice” despite knowing better (normative conformity) or they may accept the others' opinion as a valid source of information (informative conformity). Neuroimaging studies can help to distinguish between these two possibilities. Here, we present a functional magnetic resonance imaging (fMRI) study on memory conformity in a real group situation. We investigated the effects of group pressure on activity in hippocampus and anterior cingulate cortex (ACC) which likely support informative and normative memory conformity, respectively. Furthermore, we related the single nucleotide polymorphism (SNP) rs4680 [called Catechol-O-methyltransferase (COMT) Val158Met] on the gene coding for COMT to both behavior and fMRI activation. Homozygous Met-allele carriers (Val−) behaved more conformist than carriers of at least one Val-allele (Val+). In the neuroimaging data, we compared trials in which subjects were confronted with a majority of incorrect group responses to trials in which they were confronted with a majority of correct group responses. We found increased hippocampal activity when the majority of the group was correct, possibly indicating retrieval processes. Moreover, we observed enhanced activity in the ACC when the majority of the group was incorrect, suggesting that conformity was mostly normative. Most interestingly, this latter effect was more pronounced for Val− as compared to Val+ participants. This offers a speculative explanation for the higher behavioral levels of social conformity in Val− allele carriers, because their subjectively perceived conflict in the presence of an incorrect group majority may have been higher. Overall, this study demonstrates how the mechanisms leading to complex social behavior such as conformity can be studied by combining genetic analyses and fMRI in social neuroscience paradigms.

## Introduction

Historians now agree that during the Cuba missile crisis in 1962, Soviet naval officer Vasili Arkhipov likely prevented a catastrophic nuclear war, when he refused to give the necessary third approval for the launch of a nuclear-tipped torpedo in response to depth charges employed by the US navy—withstanding pressure from both the captain of the submarine and the political officer in charge. With this bold act of non-conformity during a period of insecurity and lack of relevant information (the submarine had not had access to radio news for several days), Arkhipov not only saved millions of lives, but his behavior also raises the question why some people give in to group pressure, while others do not.

In his groundbreaking work in the 1950's, Solomon Asch demonstrated that peer pressure can lead normal, well-educated participants to give blatantly false judgments about the length of lines if they are in accordance with a group of other participants, who were—unbeknownst to the subjects—confederates of the experimenter and instructed to give false responses in some trials (Asch, 1951). Asch also showed that roughly one quarter of participants were susceptible to such extreme manipulations in a consistent manner (Asch, 1956). When people conform to the opinion or behavior of a group, they may do so for a variety of reasons. Their intention may be to “play nice” and gain social approval (normative conformity), so they go along with the group despite knowing better. Alternatively, they may view the group's opinion as a valid source of information and incorporate it into their own thinking or memory (informative conformity) (Roediger et al., 2001; Cialdini and Goldstein, 2004).

Numerous conformity experiments have been designed and conducted to test the ways in which conformity affects judgment and behavior of humans. While Asch focused on conformity for the evaluation of simple perceptual stimuli, other studies investigated the impact of social influence on higher cognitive functions. Some recent studies demonstrated that responses from other participants affected subjects' recognition memory performance in a way that matched the direction of the previous group opinion (Wright et al., 2000; Reysen, 2007; Axmacher et al., 2010a). This suggests that group opinion can actually implant false memories. In situations like group learning or during eyewitness testimonies, these newly learned “memories” can become disastrous for oneself (in an important exam) or for someone else (in an eyewitness testimony) (Wells and Olson, 2003; Wright et al., 2009).

While behavioral studies have difficulty distinguishing whether informative or normative reasons cause conformist behavior, functional magnetic resonance imaging (fMRI) of the underlying neural processes may help to dissociate these processes. Normative conformity should be associated with activation in conflict-related brain areas such as the anterior cingulate cortex (ACC), while informative conformity should involve regions that are crucial for memory processes such as the hippocampus (Henke, 2010; Ranganath and Ritchey, 2012). However, there are only a few fMRI studies that investigated the neural mechanisms of conformity (Klucharev et al., 2009; Edelson et al., 2011). In the study by Edelson et al. (2011), participants had to complete a memory test about an eyewitness documentary first without manipulation (baseline accuracy), then with manipulation (false responses of other participants, immediately before MRI session), and finally again without manipulation but aware of the previous manipulation. To distinguish between normative and informative conformity, they established three conditions based on the behavioral data: persistent memory errors (indicative of informative conformity), transient memory errors (indicative of normative conformity), and non-conformity trials. Their whole-brain analyses revealed indeed that increased activation of the right hippocampus were related to informative conformity. In addition, bilateral dorsal ACC activation was associated with normative conformity. These results suggest that learning as well as conflict-related brain regions (Bush et al., 2000) can be involved in conformity, depending on the factors which induce conformist behavior.

But why are some people more susceptible to group influence than others? The difference might be due to a low confidence in their own opinion or in their own memory for conformist subjects, leading to an increased need for information from others—whether imagined or real (differences due to informative conformity). Alternatively, to some participants, differing or incorrect group opinions may simply be more unpleasant as they are for others, thereby increasing the normative pressure (differences due to normative conformity). These two options could be disentangled by investigating activation differences in conflict-related areas in the brain with functional brain imaging. Understanding differences with regard to conformity could be an important step toward preventing false eyewitness testimony and preventing “groupthink” (Janis, 1972), e.g., by composing groups in a way that conformist participants are counterbalanced by non-conformist ones.

Thus, the aim of this study was to assess the connection of social conformity and recognition memory, to investigate the underlying neuronal processes and to relate the findings to genetic differences. We developed a paradigm that created a realistic group scenario and still allowed us to manipulate group opinion. Groups of five participants were invited and underwent two parts of an experiment: The first was designed to maximize the feeling of being in a group and to establish an individual measure of conformity in the absence of manipulation, whereas in the second, fMRI was employed in one of the five subjects while the rest of the group continued behavioral testing. Group opinions in the second part were manipulated to increase the number of cases in which all members of the group gave an incorrect opinion (which maximizes group pressure). This allowed us to investigate cases of cognitive conflict and subsequent response switching with functional MRI. We focused our analyses on a crucial memory-related brain region (the hippocampus), which likely supports informative conformity (i.e., the implantation of false memories), and a region linked to conflict processing (the ACC), which is probably crucial for normative conformity (i.e., conformity due to a perceived conflict of the group majority and the correct response).

In buccal swabs taken from participants, Catechol-O-methyltransferase (COMT) Val158Met single nucleotide polymorphism (SNP; rs4680) was determined. The COMT gene is located on the q11.2 band of human chromosome 22. A G to A transition of this pleiotropic polymorphism leads to an amino acid exchange from Valine to Methione modulating thermostability and thus effectiveness of COMT, which regulates synaptic availability of dopamine and is highly expressed in the prefrontal cortex and hippocampus (Tunbridge et al., 2006; Mier et al., 2010). The 158Met-allele is associated with a three- to four-fold lower enzyme activity of COMT compared to the 158Val allele (Lachman et al., 1996). In addition, this polymorphism has been shown to modulate activation in the ACC (Blasi et al., 2005). There are three reasons why one would expect the COMT polymorphism to play a relevant role in conformity: First, it modulates neuronal activity in regions that, as discussed above, have been implicated in the neuronal processing of informative and normative conformity, i.e., the hippocampus and ACC, respectively. Second, COMT plays a role in reward and punishment (Tunbridge et al., 2006), making it an intuitive choice for the study of normative pressure, because social disapproval can serve as a powerful aversive stimulus in operant conditioning. Indeed, a previous study demonstrated higher ACC activation in response to painful stimuli for homozygous Met-allele carriers (Mobascher et al., 2010). In addition, an fMRI study by Klucharev et al. (2009) found neuroimaging evidence that reinforcement learning signals are relevant for conformist behavior. Accordingly, it has recently been hypothesized that homozygous Met-allele carriers should display more conformity due to increased sensitivity to cues that signal social disapproval (Falk et al., 2012). Third, homozygous Met-allele carriers have been associated with increased anxiousness (Montag et al., 2008, 2012) and higher risk for anxiety disorders (Domschke et al., 2004; Woo et al., 2004). Thus, this group seems more likely to seek approval by acting conformist in a group setting.

Our experimental approach is based on previous neuroimaging findings which implicate the hippocampus and ACC in conformity processing, but goes one step further by investigating differences between participants which may arise from their genetic makeup.

In the analysis of our data, we first sought to demonstrate the validity of our paradigm. This included showing that group responses induce memory conformity in a realistic group setting (i.e., in Part 1 of our study, Hypothesis A1), that experimentally manipulated group responses also induce memory conformity (i.e., in Part 2 of our study, Hypothesis A2), that this memory conformity is correlated between Part 1 and Part 2 of our study (Hypothesis A3), and that conformity occurs despite self-reported non-conformity (Hypothesis A4). Based on the literature described above, we then predicted that homozygous Met-allele carriers are more conformist than carriers of at least one Val-allele (Hypothesis B1). With regard to our imaging data, we expected that conformity-related activation of ACC or hippocampus indicates normative or informative conformity (Hypothesis C1). Lastly, we expected that a genetic × BOLD activation interaction reveals the basis for increased conformity in homozygous Met-allele carriers (Hypothesis D1).

## Materials and methods

### Participants

One hundred and forty healthy participants (81 female) took part in the experiment, 28 of whom were allotted to undergo fMRI (17 women) while the others served as a control group and were only tested behaviorally. Four non-fMRI participants were not considered in our analysis because they were recruited from the lab personnel and spontaneously filled in for subjects who did not appear to their appointment. Three additional non-fMRI subjects had to be excluded due to missing personal information (age). The mean age of the participants was 24.5 ± 3.5 years (mean ± standard deviation) (*N* = 133). Exclusion criteria were: contraindications for fMRI scanning (claustrophobia, metal implants, pregnancy, tattoos, age <18 years, neurological and psychiatric diseases, reduced state of health, and drugs affecting the central nervous system), and attendance to any experiment during the past 6 months in which faces were presented. The subjects were recruited through a public announcement over the job exchange of the University of Bonn. All subjects provided written informed consent and received monetary compensation for their participation. The study was approved by the ethics committee of the medical faculty of the University Bonn.

### Materials

Stimulus material consisted of images of unfamiliar female (50%) and male (50%) faces with a neutral emotional expression. In the first part of the experiment, 100 grayscale pictures of faces were presented with a black frame so that only the face (devoid of hair and neck) was visible. In the second part of the experiment, 300 different unknown grayscale faces were presented (without frame). The images were drawn from a large internal database. Between encoding and retrieval, subjects saw pictures of landscapes to avoid recency effects due to short-term maintenance.

### Procedure

Participants were invited in groups of five. Several days prior to their appointment, they received information about the experiment via email or regular mail. They were aware that they would be invited in groups and were given a detailed schedule of the experiment. However, they were told that the study would be on memory for faces and were neither informed that they would be taking part in a conformity study nor that group responses would be manipulated in the second part of the experiment. This deception was unavoidable for the experiment, as awareness of the scientific goal or the experimental manipulation would very likely have prevented any conformity from occurring, thus rendering the entire experiment futile. No major distress was caused by this deception and subjects were informed about the true intent of the study at the conclusion of the experiment, at the earliest possible time. Participants received this information in private and were given opportunity to retract their consent, in which case their data would have been deleted.

When all subjects had arrived on the day of the experiment, digital photographs were taken from the faces of all five participants with participants' consent. These photographs were deleted after the end of the experiment. The pictures were used in Part II as thumbnail pictures to indicate which participant had given which response with the goal of lending further credibility to the cover story. Oral swabs were taken from each subject to determine genetic polymorphisms. Participants provided written informed consent for determination of genetic polymorphisms.

**Figure 1:**
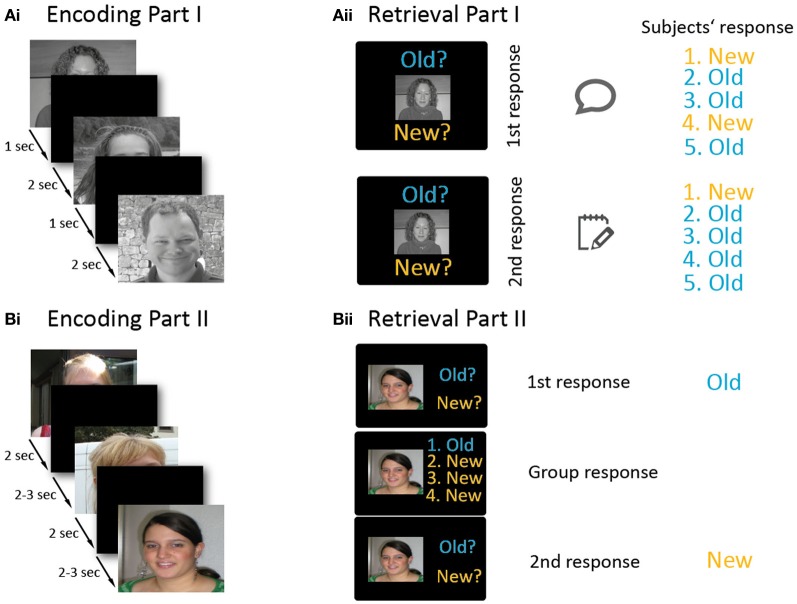
**Overview of the experimental paradigm.** The experiment involved two parts **(A** and **B)**, and each part consisted of an encoding **(Ai,Bi)** and a retrieval phase **(Aii,Bii)**. Part I **(A)** was conducted in a non-manipulated group setting. During the encoding phase **(Ai)**, participants memorized 50 stimuli which were consecutively projected onto a screen. In the retrieval block **(Aii)**, old and new stimuli were presented. For each stimulus, participants first indicated their old/new response by holding up a response tag. After viewing the responses of the other participants, they wrote down their final decision onto a sheet (which was invisible to the other participants). In part II **(B)**, each participant performed the paradigm on an individual laptop, except for one participant who conducted the paradigm inside an MRI scanner. During encoding **(Bi)**, 150 stimuli were presented. In the retrieval part **(Bii)**, subjects first decided whether a stimulus was old or new. Afterwards, the (manipulated) responses of the other four subjects were presented next to their photographs, and then participants had to indicate their—possibly revised—old/new decision. Please note that the faces presented in this figure are substitute photographs and—unlike the stimuli used in the experiment—the hair and neckline have not been masked here.

The experiment consisted of two parts. Both parts were divided into an encoding and a retrieval phase (Figure 1). Part I lasted half an hour and part II one hour. During part I, participants were seated around a table facing each other. During encoding, 50 stimuli were projected onto a large screen (Figure 1Ai). The presentation time of each stimulus was 1 s, followed by an inter-stimulus-interval of 2 s. The participants were instructed to try to memorize the stimuli. A short break with 10 landscape stimuli followed (each presented for 4 s, inter-stimulus-interval of 4 s). In the retrieval phase, all 50 previously presented faces (old items) plus 50 new faces were presented consecutively (Figure 1Aii). In each trial, a face was presented and subjects had to decide whether it was “Old” or “New” by selecting one of two response tags saying “Old” or “New.” The experimenter wrote down these first responses of the test subjects. Subjects were instructed to look at the others' replies, and then to note their final “Old” or “New” decision on a response sheet. These sheets were later used to compare first with second responses and to identify changes in decisions. In each trial, the stimulus remained on the screen until every group member had written down his or her response. Then the next trial began with an inter-stimulus-interval of 2 s. In 16% of the trials, subjects were asked to record their response immediately on their response sheets without first presenting their answer to the other four subjects. These trials served as baseline trials to measure individual recognition memory in absence of group influence. Part I of the experiment was conducted to generate an authentic group setting in order to make participants believe in the group situation and thereby minimize doubts for part II, in which participants' responses were actually manipulated.

After the first part, one of the five test subjects was randomly chosen (by drawing lots) to conduct the second part of the experiment in the MRI scanner. The other four subjects conducted the identical experiment on individual laptops in the original room. They were separated by partition panels, thus prohibiting contact between each other. Part II was constructed similar to the first part. Again, participants were instructed to memorize faces during the encoding part and asked for “Old”/“New” responses in the retrieval part.

The test instructor told the participants that—similar to Part I of the experiment—the Old/New responses would be visible to the other participants on their laptop screen via a local area network (LAN). The change in the setup-up was explained to be due to one subject now being scanned, as (s)he would have to see the others' reply on a screen inside the scanner. Subjects were instructed not to talk to each other, ostensibly because conditions were supposed to be as similar as possible to those of the subject inside the scanner, who could not hear the others either. In fact, this instruction was necessary to avoid spontaneous comments once the experiment started. The “LAN” consisted of clearly visible network cables that were leaving every laptop PC, but was, in fact, a sham network. All replies that were presented to the subjects as the responses of their peers during part II were manipulated.

The intention of part II was to investigate conforming behavior, and its neural correlates. The second part was again divided into an encoding and a retrieval phase, separated by a break (see Figure 1B). In the encoding phase, 150 stimuli were displayed on the laptop screens and, in the MRI, by video goggles. During encoding, stimuli were presented for 2 s, with an ISI of 2.5 s (in the MRI jittered between 2 and 3 s). Subjects pressed keys after presentation of each face indicating whether they found a stimulus likeable or not (Figure 1Bi). Mapping of the keys for the likeable/non-likeable answer was counter-balanced across participants. These 150 trials were followed by presentation of 10 landscape pictures to avoid recency effects due to short-term memory maintenance (presentation time for landscape stimuli: 5 s, inter-stimulus-interval: 6.5 s, in the MRI jittered between 6 and 7 s).

During the retrieval phase, each trial (total: 300 trials) was divided into two sub-parts (Figure 1Bii). In the first sub-part, a new or old picture was shown (2 s), and each subject indicated whether the presented picture was old or new. This was done by laptop keys or via buttons in the MRI. Participants were told that their responses would be visible to the other participants. This was followed by an interval of 1 s (in the MRI jittered between 0.5 and 1.5 s) during which a fixation cross was presented. In the second sub-part, the apparent responses of the other four attendees (which were in fact generated by the computer) were presented on the screen beside the thumbnail photographs of the participants that had been taken at the beginning of the experiment and participants could type in their final decision. The second sub-part together lasted 6 s. Finally, an interval of 1 s (jittered in the MRI: 0.5–1.5 s) followed before the next trial started. In 16% of all trials, no responses of the other participants were presented. These trials served as a baseline. Mapping of the keys for responding was counter-balanced across participants.

The manipulated responses were distributed in a way that favored extreme group opinions (in one third of trials all group members gave an incorrect response, in one third all group members gave a correct response, one sixth of trials were baseline trials and the remaining trials were evenly distributed to 1 correct, 2 correct, or 3 correct group responses). However, in the first 10 retrieval trials in part II, the manipulation was set up so that a majority of the group members gave correct responses. This was done to increase credibility of the setting. These first 10 trials were subsequently discarded from further analyses. After the second part of the experiment, we assessed the degree to which participants perceived group pressure and their own conformity and asked them to respond to the following questions: (1) How do you judge your own accuracy?; (2) How much did you feel biased by the others' responses?; and (3) How often did you change your response, and why did you change your response in these cases?

The experimental paradigm was presented using Presentation software (www.neurobs.com) on regular 14″ screen laptop PCs. Inside the scanner, the stimuli were presented using video goggles (Nordic Neuro Lab, Bergen, Norway).

### Behavioral analysis

In the analysis of the behavioral data, we analyzed the accuracy of the first and second response as a function of the group responses. We also analyzed switches between the first and second response, again as a function of group responses. One-Way repeated measures ANOVAS were set up with group behavior as independent variable with five levels (0, 1, 2, 3, or 4 correct responses in the rest of the group). Switches were analyzed separately for “correct switches” (switches from an incorrect first response to a correct second response) and for “incorrect switches” (switches from a correct first response to an incorrect second response), and were given as relative frequencies for every subject and condition—for example, correct switches are possible only when the first response was incorrect in the first place. Thus, we looked at the percentage of switches with regard to all cases in which switches were possible.

To establish a measure of individual conformity, we calculated a logistic regression between the number of correct responses in the group (predictor) and the second response given by the participant (criterion) across trials. This was done separately for part I and part II. A high (positive) beta value in this regression means that the second response can be well predicted by the responses previously given by members of the group and is thus a marker for the amount of conformity that an individual subject displays.

### Functional magnetic resonance imaging

Data were recorded with a Siemens Avanto 1.5T scanner (Siemens, Erlangen, Germany). We collected 31 axis slices with T2^*^-weighted, gradient echo EPI scans (slice thickness: 3 mm; matrix size: 64 × 64; field of view: 192 × 192 mm; repetition time: 2500 ms; echo time: 45 ms). Thereafter, we acquired a 3D-sagittal T1-weighted MPRAGE sequence for each subject for anatomical localization (number of slices: 160; slice thickness: 1 mm; inter-slice gap: 0.5 mm; voxel size: 1 × 1 × 1; matrix size 256 × 256; field of view: 256 mm; echo time: 3.09 ms; repetition time: 1660 ms).

### Data analysis

Preprocessing was performed using SPM 5 (www.fil.ion.ucl.ac.uk/spm/) and included the standard pre-processing steps realignment, unwarping, normalization, and smoothing with an 8-mm Gaussian kernel. A general linear model (GLM) was set-up that fitted the convolution of multiple regressors with a canonical hemodynamic response function in order to yield parameter estimates for each condition that was entered into the model. Twenty-eight subjects were included in our analysis of fMRI data. Two different models were calculated to investigate different aspects of conformity.

In the first model, three different regressors were fitted to the onset of the second response in each trial following: (1) a predominantly correct group response (3 or 4 group members correct); (2) a predominantly incorrect group response (0 or 1 group members correct); and (3) a neutral or absent group response. Note that these regressors are independent from the response of the participants. This model reflects the conflict that might arise in case of predominantly incorrect group responses. In addition, movement was modeled with a set of six continuous regressors and a linearly increasing regressor was added to account for scanner drifts. The beta weight maps of correct and incorrect group responses were then contrasted against each other and the resulting differences tested in a second level model. Both activation associated with correct group opinions (correct > incorrect group response) and activation associated with incorrect group opinions (incorrect > correct group response) were assessed.

In the second GLM, we aimed at a more detailed representation of the experimental conditions (and the putative cognitive processes). We designed a model with four factors (2 × 2 × 2 × 3 levels) which resulted in a total of 24 regressors. Onset for all regressors was the time when participants were asked to provide their 2nd response. The first factor of the model was whether an item was old or new; the second factor modeled whether a subject switched his/her first response or stayed with his/her first response; the third factor differentiated whether the final response was correct or incorrect and the last factor referred to subjects' accordance with the group (in accordance, not in accordance, group neutral/baseline trial). Five additional regressors were included modeling activity during participants' initial response: (1) trials with no response, (2) hits in the first response, (3) misses in the first response, (4) false alarms in the first response, and (5) correct rejections in the first response. Again, movement and scanner drifts were taken into account with a set of nuisance regressors. The most interesting contrast in this model was to look at switches from a correct to an incorrect response following predominantly incorrect group responses (conformity) as compared to staying with a correct response despite a predominantly incorrect group response (resistance).

We performed region-of-interest (ROI) analyses of activity in the left and right hippocampus and in the left and right ACC, as these structures are known to be involved in learning-related tasks and conflict processing, respectively. We extracted AAL template (Tzourio-Mazoyer et al., 2002) based ROIs using WFU pickatlas (http://fmri.wfubmc.edu/cms/software). We also used MNI coordinates determined in the study by Edelson et al. (2011) for small volume FWE-correction by building a sphere with 10 mm radius around the respective coordinates (right hippocampus: 28/−22/−12, left ACC: −12/22/42 and right ACC: 8/20/46).

### Genotyping

Participants provided buccal cells for genotyping. Genotyping was performed by real time polymerase chain reaction (RT-PCR) on a Light Cycler System by Roche Diagnostics, Mannheim, Germany. DNA extraction was performed with a standard commercial DNA extraction kit (MagNA Pure LC DNA isolation kit; Roche Diagnostics, Mannheim, Germany). The COMT Val158Met polymorphism was determined with a protocol as described in Reuter et al. (2011).

The COMT Val158Met SNP could be determined in 101 of 133 participants, because DNA was only available in a subsample of the participants. The genotype frequencies of COMT Val158Met were as follows: Val/Val: *n* = 20, Val/Met: *n* = 58, Met/Met: *n* = 23 and did not deviate from the Hardy–Weinberg equilibrium (*X*^2^ = 2.26, *df* = 1, n.s.). For the 28 subjects undergoing fMRI, we were able to determine the SNP in 23 subjects (Val/Val = 5, Val/Met = 10, Met/Met = 8, no deviation of Hardy–Weinberg equilibrium *X*^2^ = 0.31, *df* = 1, n.s,). For all analyses, we combined Val/Val and Val/Met carriers (Val+) and compared them against Met/Met carriers (Val−). This was done because it has been shown that especially Val− subjects are afflicted with increased anxiousness (Montag et al., 2008).

## Results

### Descriptive statistics

Subjects who had an accuracy rate <50%, did not give an answer in more than 10% of all trials or failed to complete the final questionnaire were excluded from all further analysis, which left us with 104 participants for the analysis of part I and 121 participants for the analysis of part II.

In the first part, we found that 68.1 ± 0.8 (mean ± standard error of the mean) of all faces (total: 100) were correctly recognized in the first response. Accuracy during the second responses (after having seen the responses given by the other attendants) was at 68.2 ± 0.7. In the baseline condition, in which no group rating was given, the accuracy was at 58.8 ± 1.4.

**Figure 2:**
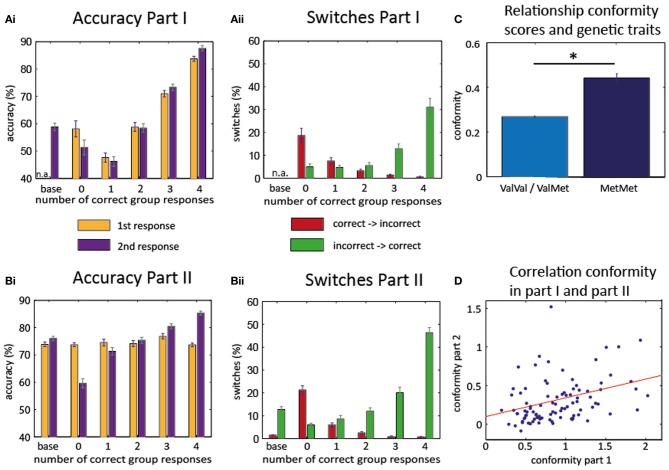
**Behavioral results.** Accuracy part I **(Ai)** and part II **(Bi)**: increase of the accuracy rate for the 2nd response as more participants give a correct response. Switches part I **(Aii)** and part II **(Bii)**: increase of correct switches (from incorrect to correct, green) when more participants give a correct response. Decrease of incorrect switches (from correct to incorrect, red) with more correct responses in group. Baseline trials in the first part consisted only of the second (written) response of each participant. Therefore, accuracy of first response and switches could not be evaluated for these trials (n.a.). **(C)** Association of conformity scores with genetic polymorphisms. Homozygous Met/Met-allele carriers show higher conformity scores than carriers of at least on Val-allele. **(D)** High correlation between conformity scores of the first and second part: subjects who behaved conformist in part I also exhibited conformity in part II. ^*^T_(99)_ = −2.4547, *P* = 0.0158.

In the second part, accuracy for the first responses was 74.0% ± 0.6% and for the second response 73.6% ± 0.7%. During baseline trials, 76.0 ± 0.8 items were remembered correctly (see Figure 2Bi).

### Hypothesis A1: group responses induce memory conformity in a realistic group setting

As can be seen in Figure 2Ai, individual accuracy in the second and final response varied with the number of correct responses of the other group members [*F*_(4, 280)_ = 58.831, *p* < 0.001; only 71 subjects were considered for this ANOVA because group opinion was not experimentally manipulated in part one and some subjects did not experience any trials in which all other group members gave an incorrect response]. There was a significant linear effect across the group [*F*_(1, 70)_ = 128.632, *P* < 0.001] with a steady rise in accuracy for more correct group opinion. However, the same pattern was already evident for the first responses, i.e., before any group responses had been seen [effect of subsequent group response: *F*_(4, 280)_ = 41.945, *P* < 0.001; linear effect *F*_(1, 70)_ = 88.158, *P* < 0.001]. This suggests that the opinions of the group members were not independent from each other in the first part and group opinion probably reflected item difficulty, i.e., trials for which all members of the group gave a correct response were in general easier to remember.

Next, we calculated the number of switches (i.e., changes from the first to the second response) depending on the number of correct group responses (Figure 2Aii). We found that the probability for switching from an incorrect first response to a correct second response (“correct switches”) differed depending on the number of correct responses given by the other participants [*F*_(4, 280)_ = 20.674, *p* < 0.001], and that there was a linear effect of the number of correct group responses [*F*_(1, 70)_ = 29.125, *P* < 0.001]. As expected, more correct switches occurred when more members of the group gave a correct response. Even more importantly, the number of incorrect switches (switching from a correct first response to an incorrect second response) also differed for the number of correct group responses [*F*_(4, 280)_ = 17.595, *P* < 0.001] and linearly decreased with the number of correct responses of the other participants [*F*_(1, 70)_ = 26.385, *P* < 0.001]. The likelihood to change from an incorrect to a correct response was higher than the likelihood to change from a correct to an incorrect response, but the modulation by the group responses was not different [Two-Way repeated measures ANOVA with “correct vs. incorrect switches” as first factor and “number of group responses that differed from own first response” as second factor; main effect for the first factor (*F*_(1, 70)_ = 7.765, *P* = 0.007); main effect for the second factor (*F*_(4, 280)_ = 38.870, *P* < 0.001); interaction not significant (*F*_(4, 280)_ = 1.141, *P* = 0.337)].

### Hypothesis A2: manipulated group responses also induce memory conformity

In part II, the accuracy rate during the first response should be independent of the manipulated “responses” of the other group members. Again, the accuracy rate during the second response strongly differed with the number of group members who gave correct responses [*F*_(4, 480)_ = 83.863, *P* < 0.001], and increased linearly [*F*_(1, 120)_ = 150.721, *P* < 0.001].

Unexpectedly, the first response also differed for the number of correct responses given by the group [*F*_(4, 480)_ = 2.906, *P* = 0.021]. However, this effect was much weaker than in Part I and was due to a rise in accuracy for trials in which 3 out of 4 group members gave correct responses. *Post-hoc t*-tests showed that accuracy in this condition differed significantly from the accuracy in two other conditions (significant difference to trials with 1 and 5 correct group responses: *p*_corr_ = 0.023 and *p*_corr_ = 0.005, respectively; Bonferroni-corrected), while accuracy in the remaining conditions did not vary (all *p*_corr_ > 0.198). Importantly, there was no linear effect of the number of correct group responses on the accuracy of the first response [*F*_(1, 120)_ = 1.688, *P* = 0.196].

A Two-Way RM-ANOVA with first vs. second response as first factor and group responses as second factor confirmed the different effects of the group on first and second responses. This analysis showed that the main effect for first vs. second responses was not significant [*F*_(1, 120)_ = 0.341, *P* = 0.56], indicating that the overall accuracy did not differ between first and second responses; the main effect of number of correct group responses was significant again [*F*_(4, 480)_ = 38.704, *P* < 0.001]; most interestingly, the interaction was also significant [*F*_(4, 480)_ = 124.156, *P* < 0.001], confirming that the modulation by group response was different in the first and second responses.

The number of switches (Figure 2Bii) from initially incorrect to correct responses again depended on the number of correct group responses [*F*_(4, 480)_ = 91.393, *P* < 0.001] in a linear fashion [*F*_(1, 120)_ = 203.665, *P* < 0.001]. A similar pattern was evident for the switches from initially correct to incorrect responses [*F*_(4, 480)_ = 98.017, *P* < 0.001; linear effect *F*_(1, 120)_ = 120.664, *P* < 0.001]. Thus, subjects revealed a clear effect of social conformity even if it meant giving up an initially correct opinion. The likelihood to change from an incorrect to a correct response was again higher than the likelihood to change from a correct to an incorrect response, but here the modulation by the group responses was indeed different for correct and incorrect switches [Two-Way repeated measures ANOVA with the “correct vs. incorrect switches” as first factor and “number of group responses that differed from own first response” as the second factor; main effect for the first factor (*F*_(1, 120)_ = 197.495, *P* < 0.001; main effect for the second factor *F*_(4, 480)_ = 122,493, *P* < 0.001); interaction *F*_(4, 480)_ = 26.541, *P* < 0.001]. The significant interaction in this ANOVA likely reflects a sharper increase in the number of switches for the correct switches as compared to the incorrect switches.

### Hypothesis A3: memory conformity is correlated between Part 1 and Part 2

Next, we quantified the amount of social conformity in individual subjects (by calculating a regression between group response and second response; see “Materials and Methods”). Conformity scores in part I were higher than in part II [*T*_(93)_ = 14.201, *P* < 0.001, based on 94 subjects, whose data were analyzed for both parts], which would be expected because the group response in part I is not manipulated and therefore reflects how easily a particular item can be remembered (therefore, group effect and individual memory tend to influence the second response in the same direction). However, conformity scores in the second part (0.29 ± 0.02 mean ± s.e.m.) were still significantly higher than zero [*T*_(120)_ = 10.9419, *P* < 0.001]. Most importantly, we observed a significant inter-individual correlation between conformity scores in part I and part II [Pearson correlation: *R*_(92)_ = 0.37, *P* = 0.0002], indicating that conformist participants in part I tended to be conformist in part II as well (Figure 2D).

In addition, we tested if memory conformity was due to poor memory performance. The analysis revealed that participants with poor memory performance showed a higher level of conformity [*R*_(119)_ = −0.3157, *P* < 0.001].

### Hypothesis A4: conformity occurs despite self-reported non-conformity

Participants who stated in the final questionnaire that they had been influenced by the group (stating “sometimes,” “usually,” or “always” influenced by the group) had indeed higher conformity than those participants who reported that they had not been influenced by the group [stating “never,” “once,” or “usually not” influenced by the group, *T*_(116)_ = 6.48, *P* < 0.001, 3 subjects dropped out due to missing values]. However, these “low influence” participants still showed significant conformity [*T*_(45)_ = 6.66, *P* < 0.001].

### Hypothesis B1: homozygous Met-allele carriers are more conformist

To find out whether genetic variants have an impact on conformity, we related the individual conformity scores to the genetic markers. This analysis revealed that Met/Met-allele carriers showed higher conformity values than the combined group of Val/Val and Val/Met carriers [mean conformity Met/Met = 0.44, mean conformity Val/Val and Val/Met = 0.2691, two-sample *T*-test *T*_(99)_ = −2.4547, *P* = 0.0158, see Figure 2C]. Likewise we related the genetic markers to the individual memory performance and found that there was no association [*T*_(99)_ = 1.3251, *P* = 0.1882]. Next, we tested whether the influence of COMT on conformity was independent of the effect of COMT on memory performance. Thus, we set up a univariate ANCOVA with COMT Val158Met (Val±) as independent variable, memory performance as covariate and conformity as dependent variable. This showed that the effect of COMT was still significant [*F*_(1, 98)_ = 4.56, *P* = 0.03].

### Hypothesis C1: conformity-related activation of ACC or hippocampus indicates normative or informative conformity

In a *first GLM*, We investigated the influence of group responses on neural activity. We compared trials in which the group response was predominantly correct (3 or 4 correct) with trials in which the group response was predominantly incorrect (0 or 1 correct). Two ROI analysis approaches were pursued. First, we averaged activity in anatomical masks in bilateral hippocampus and bilateral ACC based on AAL (Tzourio-Mazoyer et al., 2002). This analysis revealed greater activation within bilateral hippocampus when the group was correct [Left: *T*_(27)_ = 3.1805, *p* = 0.004; Right: *T*_(27)_ = 2.5586, *p* = 0.016]. There were no significant differences for the left or the right ACC [left: *T*_(27)_ = 1.32, *P* = 0.19, right: *T*_(27)_ = −0.2, *P* = 0.84]. Second, to investigate more spatially restricted activity clusters, we conducted a small-volume correction analysis in ROIs defined by spheres around the MNI coordinates of the Edelson et al. (2011) study. Again, correct group responses were associated with increased activation in a cluster in the right hippocampus [*T*_(27)_ = 4.94, *P*_FWE_ = 0.002]. In the reverse contrast, we found a marginally significant cluster in the right ACC [*T*_(27)_ = 3.46, *P*_FWE_ = 0.049]. These results can be interpreted as an involvement of possibly memory-related hippocampus activity during trials in which the majority of the group is giving a correct response and recruitment of a prototypical conflict-related region when the majority of the group is giving an incorrect response.

**Figure 3:**
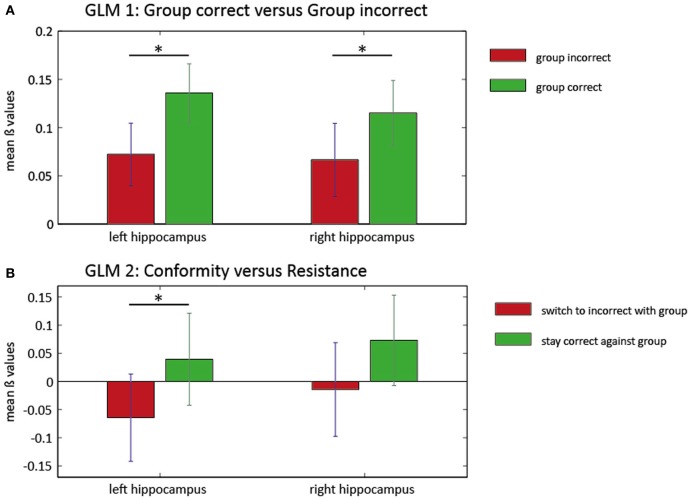
**ROI analysis in the hippocampus for two general linear models.** Mean beta values and SEM of the ROI analysis in left and right hippocampus for two GLMs. In the first model **(A)**, the group influence on BOLD responses within the hippocampus is illustrated. There is significantly higher hippocampus activation (left and right) when the group is predominantly correct (green). Left panel: ^*^T_(27)_ = 3.1805, *P* = 0.004; right panel: ^*^T_(27)_ = 2.5586, *P* = 0.016. The second GLM **(B)** reveals increased activity in the left hippocampus when subjects resist the group (green) compared with trials where conforming responses were given (red). There was no significant increase of activity in the right hippocampus for this contrast. Left panel: ^*^T_(23)_ = 2.082, *P* = 0.049.

Conforming behavior may occur when participants initially give a correct response, but are then faced with a majority of incorrect group responses. In a *second GLM*, we therefore investigated the effect of incorrect group responses on initially correct responses more specifically. We contrasted trials in which participants resisted group pressure in this situation (“resistant” trials) with trials in which they switched to an incorrect group response (“conformist” trials). Using anatomical masks from AAL, we observed increased BOLD responses in resistant vs. conformist trials in the left hippocampus (Figure 3) [*T*_(23)_ = 2.082, *P* = 0.049] but not in the right hippocampus [*T*_(23)_ = 1.309, *P* = 0.204]. No clusters were found in the ROIs based on the coordinates described by Edelson et al. (2011).

### Hypothesis D1: genetic × BOLD activation interaction reveals the basis for increased conformity in homozygous Met-allele carriers

**Figure 4:**
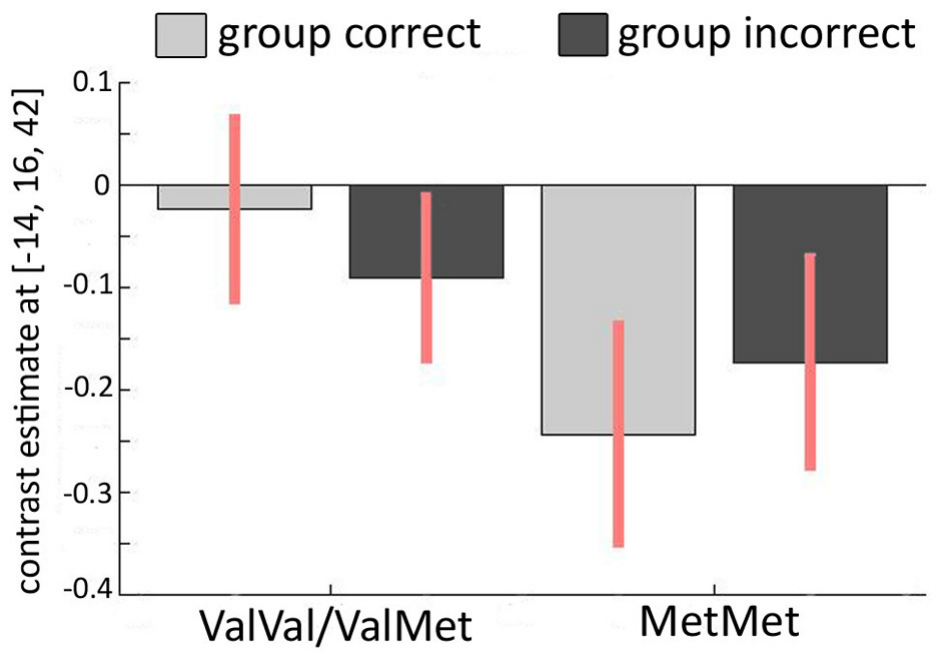
**Relationship between COMT Val158Met and imaging results.** Interaction of incorrect vs. correct group response and COMT polymorphism in the left ACC (MNI −14/16/42). The activation pattern for carriers of at least one Val-allele (Val+) is different from the pattern found in homozygous Met-allele carriers (Val−) across the two conditions in which the majority of the group is either correct or incorrect, revealing a significant interaction between genotype and brain activation.

Finally, we investigated the relationship between the COMT Val158Met polymorphism and conformity-related brain activations. In our *first GLM* (contrasting incorrect and correct group responses), we thus investigated “genotype” × “activity” interactions. Again, we analyzed both averaged activity within AAL masks and spherical ROIs around hippocampal and ACC coordinates from Edelson et al. (2011). We did not find any differences between the groups across the entire AAL-based ROIs. However, we observed significant “genotype” × “activity” interactions in two left ACC clusters within the spherical ROIs around the coordinates found in the Edelson et al. (2011) study [−4/20/40: *T*_(21)_ = 5.01, *P*_FWE_ = 0.004; −10/18/42: *T*_(21)_ = 4.46, *P*_FWE_ = 0.01; see Figure 4]. In participants with at least one Val-allele, this contrast was not significantly different from zero [*T*_(14)_ = 1.761, *P* = 0.10]. In homozygous Met-allele carriers, we found increased ACC activity during incorrect group trials, possibly due to a subjectively perceived higher degree of conflict [*T*_(7)_ = −5.067, *P* = 0.002]. Speculatively, the increased conflict-related activation in the left ACC in the Met/Met-allele carriers may explain their increased conformity that was found on a behavioral level (Figure 2C). This finding was further confirmed with a Two-Way ANOVA in which the COMT allele variants (Val+ vs. Val−) represented a between-subjects factor and group response was a within-subjects factor. Again, we found a significant interaction in the left ACC [−14/16/42: *F*_(1, 42)_ = 13.26, *P*_FWE_ = 0.041]. In the second GLM, contrasting “resistant” vs. “conformist” trials, no significant influence of the COMT Val158Met polymorphism was found around the ACC or the hippocampus.

## Discussion

In this multi-methodological study, we investigated the neuronal correlates of conformity and resistance to conformity in a paradigm that involved a realistic group situation and still allowed us to test participants in an MRI scanner while they were exposed to group pressure.

On a behavioral level, we found an increase in response switches in trials in which group opinion differed more drastically from the opinion of a participant. This is good evidence that our paradigm was successful in inducing conformity: While in the first part of the experiment, during which group opinion was not manipulated, item difficulty may have been a contributing factor to the group-induced changes in responses, this was precluded in the second part of the experiment, during which group opinion was experimentally manipulated by the computer program. Interestingly, self-reported indifference to group opinion was associated with reduced but still significant susceptibility to group responses.

We found evidence that the genetic polymorphism in COMT Val158Met had a significant impact on the susceptibility to group influence: Homozygous Met-allele carriers showed more conformity than did carriers of at least one Val-allele. This has recently been hypothesized by Falk et al. (2012), reasoning that homozygous Met-allele carriers might be more sensitive to cues that signal social reward or punishment. Homozygous Met-allele carriers also exhibit greater anxiety (Montag et al., 2008). This might predispose them to seek approval of the group and thus exhibit more conformity. On a biochemical level our results suggest that social conformity is in parts influenced by dopaminergic neurotransmission. Although it is problematic to derive dopamine levels from the information of just one SNP one might speculate that carriers of the Met/Met variant are associated with highest dopamine levels (due to lower COMT activity). Although the effect of COMT on synaptic dopamine concentration is probably most pronounced in the lateral prefrontal cortex, where dopamine clearance due to synaptically located dopamine transporters (DAT) is relatively low (Sesack et al., 1998; Lewis et al., 2001) and thus depends on COMT to a large degree, our results tentatively suggest that COMT also affects activity in the ACC, possibly by an enhanced perception of conflict. However, it is of importance to keep in mind that the dopamine levels in the synaptic cleft are influenced by a large number of other factors such as drift of dopamine out the synaptic cleft, the number of available DAT or the catabolizing enzyme monoamino-oxidase. Therefore we reiterate that our line of argumentation warrants more empirical evidence in the future. In addition, the number of participants per genotype in the imaging analysis was relatively low in this study (especially homozygous Met-allele carriers with *N* = 8). It should be higher and more balanced in future studies.

When we investigated the neuronal correlates of conformity in the fMRI data, we found that predominantly correct group responses were associated with increased hippocampal activation. The hippocampus is known for its pivotal role in human declarative memory (Squire et al., 2004). As we investigated the accuracy of group responses irrespective of participants' own responses, this increase in activation may appear surprising at first sight. However, a correct group opinion should on average be more in accordance with participants' own responses (as they were accurate in 74.0% ± 0.6% of all first responses). The increased hippocampal activity that is associated with correct group responses may therefore reflect a strengthening of existing memory traces that have been confirmed by a matching correct group response. We also found (in our second GLM) increased activity in the left hippocampus in trials in which participants resisted the group influence (i.e., stayed with their correct response despite a majority of incorrect group responses) as compared to trials in which they switched to an incorrect group-conforming response. This might be an indicator that resistance to conformity goes along with a higher confidence in one's own responses in the first place. Yet, it may also reflect an updating process in the sense that participants mark the memory for this particular item as potentially false—in general, reverse inference in fMRI studies is inherently problematic (Poldrack, 2006; Axmacher et al., 2009).

In future studies, two features should be implemented to clarify the significance of hippocampal activation further: first, an assessment of the confidence in the first response on a scale from “very sure” to “guessing.” This could help dissociating between insecurity and real conformity. In addition, a second memory test subsequent to the manipulated group phase may reveal whether the item information has indeed been updated according to the group opinion. However, it should be noted that even such measures do not allow one to unequivocally distinguish between informative and normative conformity: if a participant responds conforming to an incorrect group opinion even after a delay, this may be due to informative conformity. However, it is also possible that participants have initially responded to conform to the group despite better knowledge (normative conformity) but have subsequently come to believe that this incorrect response was actually correct (informative conformity)—memories are constantly being reconstructed (e.g., Axmacher et al., 2010b).

A second finding was that when the majority of group responses were incorrect, activity in the ACC increased. This is good evidence that in some of these trials, a conflict is detected. The increase in ACC activation was especially pronounced in homozygous Met-allele carriers, indicating that conflict might be perceived more drastically in this group. The Met-variant of the COMT gene has been associated with increased pain sensitivity (Zubieta et al., 2003) and greater reactivity of the limbic system and ventrolateral prefrontal cortex to unpleasant stimuli (Smolka et al., 2005). Together with our finding of increased ACC activation during confrontation with incorrect group responses, this might support the notion that disagreeing with a group is more unpleasant and causes greater conflict in carriers of the Met/Met-allele. This might also explain why Val− carriers exhibit more conformity on a behavioral level. We did not find any significant ACC activation in a whole-group analysis in our second model, which contrasted “resistant” vs. “conformist” trials. This may be due to two reasons: First, conflict was induced in both kinds of trials (because in both cases, the group response differed from the correct own first response) and any difference in the degree of conflict might have been too small to be detected. Second, the number of these trials was relatively low, possible resulting in a lack of power to reveal subtle differences.

In summary, the paradigm presented here enabled us to create a realistic group situation and to induce conformity even in subjects who stated that they were mostly indifferent to what the group said. The tendency to agree with a group opinion seemed to be influenced by neurobiological factors that affect dopaminergic neurotransmission and was possibly related to experiencing greater conflict when the own opinion was different from the group opinion.

### Conflict of interest statement

The authors declare that the research was conducted in the absence of any commercial or financial relationships that could be construed as a potential conflict of interest.
